# Mechanisms of transcutaneous bilateral auricular vagus nerve stimulation in treating functional dyspepsia through promoting M2 macrophage polarization

**DOI:** 10.1371/journal.pone.0328670

**Published:** 2026-08-21

**Authors:** Daye Yang, Yiran Liu, Shuangling Ou, Bingxue Liang, Long Li, Chenglin Tang, Dan Wang

**Affiliations:** 1 Department of Acupuncture-Moxibustion and Tuina, Chongqing University of Chinese Medicine, Chongqing, China; 2 College of Traditional Chinese Medicine, Chongqing Medical University, Chongqing, China; Weill Cornell University, UNITED STATES OF AMERICA

## Abstract

**Background:**

Functional dyspepsia (FD) is characterized by duodenal mucosal barrier dysfunction and chronic low-grade inflammation. Macrophage polarization imbalance, particularly the predominance of pro-inflammatory M1 over anti-inflammatory M2 phenotypes, contributes significantly to FD pathogenesis. Transcutaneous auricular vagus nerve stimulation (taVNS) has emerged as a promising non-invasive therapeutic approach for gastrointestinal disorders.

**Objective:**

This study aimed to investigate the therapeutic mechanisms of taVNS in FD treatment, specifically focusing on its effects on M2 macrophage polarization and the roles of metabolic regulator UCP2 and transcription factor GATA3 in this process.

**Methods:**

Male C57BL/6J mice (*n* = 27) and *Gata3*^∆mac^ mice (*n* = 9), totaling 36 mice were used to establish an FD model through multi-factorial stress induction and were divided into four experimental groups. taVNS treatment was administered for 2 weeks. Feeding behavior, duodenal mucosal inflammatory cytokines, inflammatory markers, macrophage polarization markers, and intestinal barrier function were assessed. *Gata3*^*∆*mac^ + taVNS mice were used to determine the transcription factor’s role in taVNS-mediated effects.

**Results:**

TaVNS treatment significantly improved feeding behavior in FD mice. At the duodenal mucosal level, taVNS markedly reduced the elevated IL-1βlevels and restored the suppressed TGF-β1 levels. TaVNS also promoted M2 macrophage polarization, as evidenced by decreased *Nos2* expression and increased Arg1 expression. TaVNS partially restored *Ucp2* and *Gata3* expression relative to FD mice, elevated the expression of tight junction ZO-1 to relieve duodenal tight junction impairment linked to barrier dysfunction, and modulated inflammatory responses (decreased TNF-α, increased IL-10). The therapeutic effects of taVNS were significantly attenuated in *Gata3*^∆mac^ + taVNS mice, confirming the critical role of GATA3 in mediating taVNS-induced M2 polarization.

**Conclusions:**

taVNS promotes M2 macrophage polarization to treat FD through mechanisms involving both metabolic regulation (UCP2) and transcriptional control (GATA3). These findings provide novel insights into the immunometabolic mechanisms underlying taVNS therapy and support its clinical application for FD management.

## 1. Introduction

Functional dyspepsia (FD) is one of the most common functional gastrointestinal disorders, defined by the Rome IV criteria as the presence of bothersome postprandial fullness, early satiation, epigastric pain, or epigastric burning sensation without structural abnormalities that can explain the symptoms [1,2]. FD affects a substantial proportion of the global population, resulting in significant healthcare burden and impaired quality of life [3]. Its pathophysiology is complex and multifactorial, involving gastric sensorimotor dysfunction, visceral hypersensitivity, immune activation, microbiota dysbiosis, and brain-gut axis dysfunction [4,5]. Emerging evidence indicates that duodenal mucosal barrier dysfunction, characterized by increased intestinal permeability and compromised tight junction integrity, plays a pivotal role in FD pathogenesis [6].

Intestinal mucosal barrier dysfunction represents a core feature of FD pathophysiology, with studies demonstrating that increased intestinal permeability is closely associated with symptom severity in functional gastrointestinal disorders [6]. Compromised intestinal barrier function leads to bacterial product and antigen translocation, triggering local and systemic immune responses [7]. Systematic studies have confirmed the presence of duodenal mucosal inflammatory cell infiltration and elevated circulating inflammatory markers in FD patients [8]. The bidirectional regulation of the brain-gut axis plays an important role in the pathogenesis of functional gastrointestinal disorders, where psychological stress and gastrointestinal dysfunction interact to form a vicious cycle [9]. Macrophages, as key components of the intestinal innate immune system, play a central role in maintaining intestinal homeostasis [10]. These cells can switch between pro-inflammatory M1 phenotype and anti-inflammatory reparative M2 phenotype according to microenvironmental signals [11]. In the pathological state of FD, macrophage polarization imbalance may lead to persistent low-grade inflammation and impaired mucosal barrier function. Therefore regulating macrophage polarization represents an important therapeutic strategy for repairing duodenal mucosal barrier damage in FD.

Immunometabolic studies have shown that metabolic reprogramming is a fundamental mechanism controlling macrophage polarization [11]. M1 and M2 macrophages exhibit distinct metabolic characteristics: M1 macrophages primarily rely on the glycolytic pathway, while M2 macrophages depend on oxidative phosphorylation and fatty acid oxidation to maintain anti-inflammatory and tissue repair functions [12]. Mitochondrial function plays a key role in macrophage metabolic reprogramming [13]. Mitochondrial uncoupling protein 2 (UCP2), as an important metabolic regulator, has been shown in recent studies to control cellular function by regulating oxidative phosphorylation and influencing the inflammatory state of macrophages [14]. UCP2 plays a significant role in glucose and lipid metabolism, with its expression finely regulated by nutritional status and metabolic signals [15], and it also plays a crucial role in regulating macrophage polarization (particularly promoting the M2 phenotype). In terms of immune cell differentiation, the transcription factor GATA3 has been shown to play a central regulatory role in the development and function of innate lymphoid cell subsets [16]. More importantly, in macrophages, GATA3 also participates in regulating their fate determination. This transcription factor-driven cell differentiation mechanism provides important insights into understanding macrophage polarization [17]. Therefore, *Ucp2*-mediated metabolic regulation and GATA3-mediated transcriptional control potentially constitute an important molecular network regulating macrophage polarization, providing new targets for FD treatment. Specifically, UCP2, as a mitochondrial uncoupling protein, plays a core role in macrophage metabolic reprogramming by modulating oxidative phosphorylation efficiency and reactive oxygen species (ROS) levels, and its expression pattern is closely related to the maintenance of the anti-inflammatory M2 phenotype. Meanwhile, the transcription factor GATA3 is not only the master regulator of Th2 cell differentiation, but recent evidence also suggests its key role in macrophage polarization, capable of directly driving the expression program of M2-related genes [18]. Based on this, we speculate that taVNS may promote the conversion of macrophages to the M2 phenotype by synergistically regulating UCP2 and GATA3, thereby repairing duodenal mucosal barrier function and improving FD symptoms.

Transcutaneous auricular vagus nerve stimulation (taVNS), as a non-invasive neuromodulation technique, holds therapeutic potential for FD due to its multi-mechanistic action aligning closely with the multifactorial pathophysiology of the disorder [19]. By activating cholinergic anti-inflammatory pathways, taVNS exerts anti-inflammatory effects while also regulating gastrointestinal motility and intestinal immune homeostasis [20]. This mechanism may indirectly modulate the duodenal immune microenvironment via central descending cholinergic anti-inflammatory circuits and promote macrophage polarization toward the anti-inflammatory M2 phenotype. Additionally, auricular vagal stimulation elevates vagal efferent output to rebalance autonomic nervous tone away from sympathetic overactivation [21–23], which facilitates gastric emptying, improves gastric accommodation, and alleviates visceral hypersensitivity [21]. Beyond peripheral gastrointestinal regulation, taVNS elicits broad central neuromodulatory effects. It modulates noradrenergic signaling within brainstem nuclei including the nucleus tractus solitarius and locus coeruleus to regulate arousal and cognitive processing [24,25]. A human behavioral and physiological study confirmed that vibrotactile taVNS improves working memory through this arousal-modulation mechanism [26]. These effects collectively address the complex symptoms of FD [27]. International consensus has established standardized reporting guidelines for taVNS research, providing important guidance for clinical translation [28]. Systematic reviews demonstrate that taVNS has good safety and tolerability in human applications [29].

## 2. Materials and methods

### 2.1 Animals and grouping

A total of 36 male mice were used in this study, including 27 C57BL/6J mice (8–10 weeks old, 20-25g) purchased from the Experimental Animal Center of Chongqing Medical University (Chongqing, China), and 9 *Gata3*^fl/fl^:Cx3cr1^CreERT2^ (*Gata3*^∆mac^) macrophage-specific conditional knockout mice (8–10 weeks old, 20-25g) obtained from Shanghai Model Organisms Center, Inc.(Shanghai, China). To induce Gata3 deletion, these mice received tamoxifen at 125 mg/kg via intraperitoneal injection every other day for 4 days before the experiments. This study exclusively utilized male mice to exclude potential confounding effects of the estrous cycle in female animals on gastrointestinal sensitivity, immune responses, and stress-related behaviors, thereby reducing experimental complexity. The mice were randomly assigned by a computer-generated random number table into four groups: normal control group (NOR), functional dyspepsia model group (FD), taVNS treatment group (taVNS), and *Gata3*^∆mac^ with taVNS treatment group (*Gata3*^∆mac^ + taVNS). All investigators conducting 3-h food intake behavioral testing, tissue dissection and sample processing, as well as subsequent qPCR, Western blot and ELISA quantitative data analysis were blinded to experimental group allocation throughout the whole experiment. For behavioral assays (3-h food intake) and ELISA measurements of duodenal mucosal cytokines (IL-1β and TGF-β1), all six mice per group were used (*n* = 6 per group). For ELISA measurements of serum inflammatory cytokines (TNF-α and IL-10), Western blot analysis of ZO‑1, and qPCR analysis of *Ucp2, Gata3, Arg1, Nos2*, and other genes, three mice per group were randomly selected from the respective cohorts (*n* = 3 per group).

All animals were housed in a specific pathogen-free environment with a 12-hour light/dark cycle at 22 ± 2°C and 55 ± 5% humidity, with free access to standard chow and water. The mice were acclimatized for one week before experimental procedures. Daily monitoring of animal welfare, including body weight, food/water intake, and general behavioral signs (e.g., posture, locomotion, and response to handling), was performed throughout the experiment. Humane endpoints were predefined as >20% loss of initial body weight, persistent hunched posture, severe lethargy, or inability to access food/water; any mouse reaching these criteria was immediately euthanized with an overdose of isoflurane followed by cervical dislocation. For any procedures requiring anesthesia (e.g., tissue collection or surgical manipulation), isoflurane inhalation (2–3% in oxygen) was used, and all efforts were made to minimize suffering; no chloral hydrate or other prohibited agents were employed. All experimental protocols were approved by the Institutional Animal Care and Use Committee of Chongqing Medical University (IACUC-CQMU-2023–0378) and conducted in accordance with the National Institutes of Health Guidelines for the Care and Use of Laboratory Animals.

### 2.2 Model establishment

**Fig 1:**
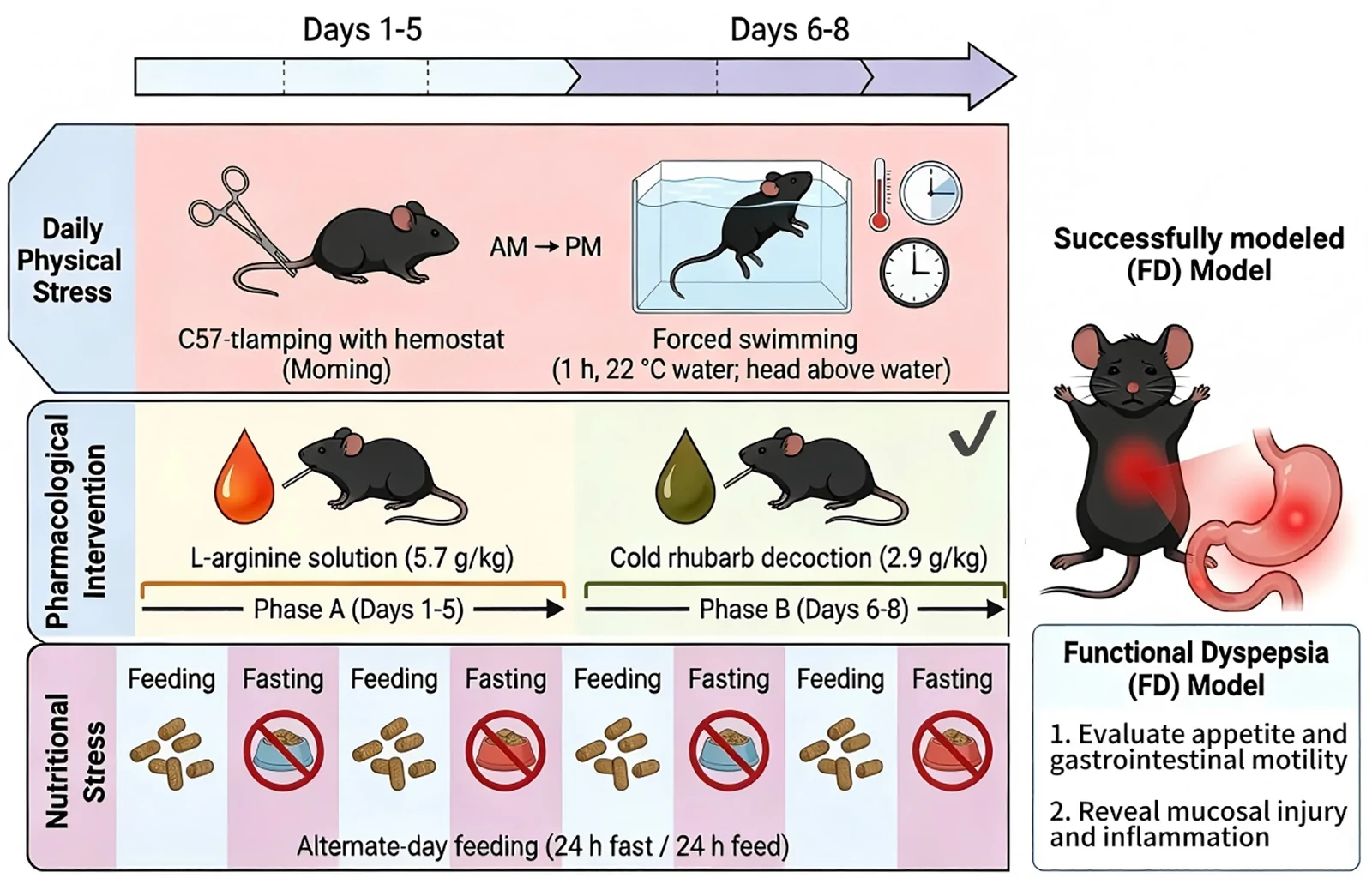
Multi-factor multi-step Functional Dyspepsia (FD) mouse model protocol.

The FD model was established using a comprehensive multi-factor approach [26],following a protocol similar to that described in prior work over 8 consecutive days. Modeling mice received daily morning tail-clamping. In the afternoon, mice were subjected to forced swimming for 1 hour in a temperature-controlled water tank (22°C) using a rectangular water tank (110 cm × 60 cm × 40 cm) filled with 10 cm depth of warm water. Immediately after swimming, mice received gavage administration of L-arginine solution (5.7 g/kg) for the first 5 days (Days 1–5). From Day 6 onwards, L-arginine was replaced with cold rhubarb decoction (2.9 g/kg) for an additional 3 days (Days 6–8), while maintaining all other procedures unchanged. Throughout the entire 8-day modeling period, mice were subjected to alternate-day feeding regimen (24-hour fasting alternating with 24-hour feeding access) starting from Day 1. This comprehensive protocol combining stress stimulation (tail-clamping, forced swimming), pharmacological intervention (L-arginine followed by rhubarb decoction), and nutritional stress (intermittent fasting) was designed to replicate the multifactorial pathophysiology of functional dyspepsia (Fig 1).

### 2.3 TaVNS intervention method

**Fig 2:**
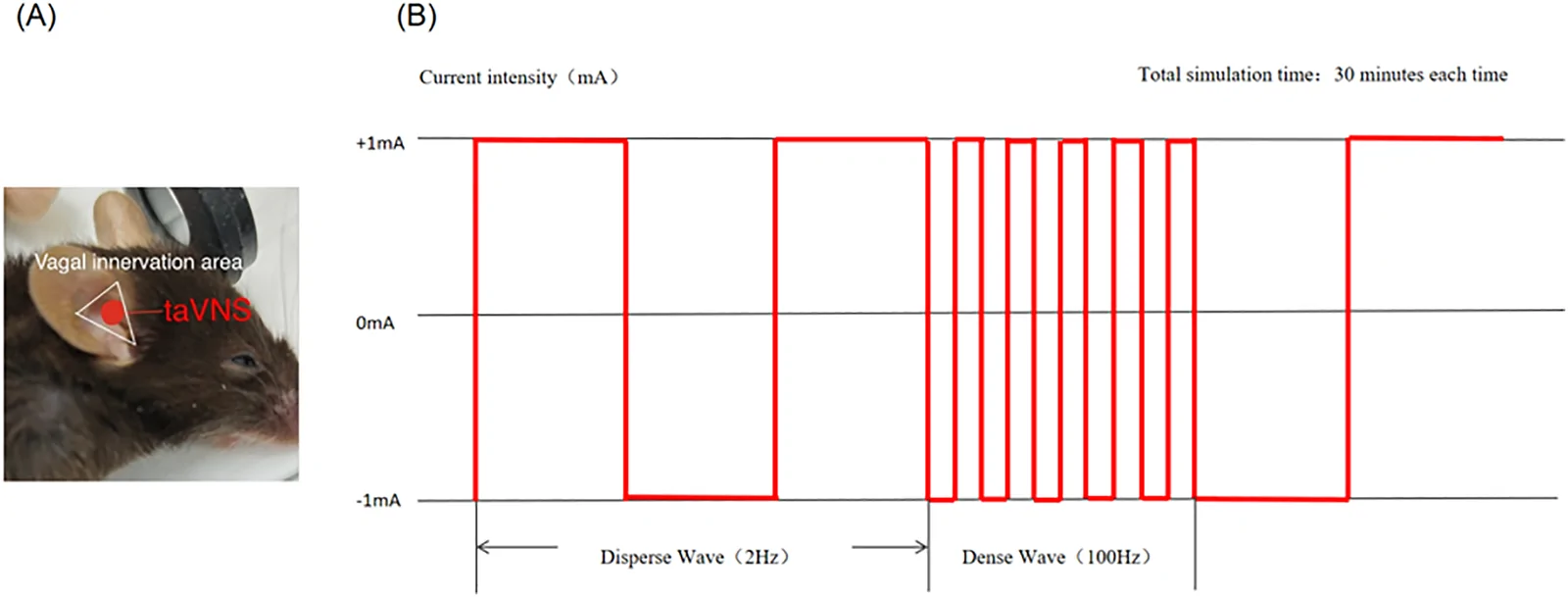
Schematic of the transcutaneous auricular vagus nerve stimulation (taVNS) protocol. (A) Position of the stimulation electrodes on the inner and outer surface of the mouse pinna. The electrodes were connected to a HANS-100A stimulator. (B) The stimulation paradigm consisted of a disperse-dense wave pattern, alternating between a low-frequency (2 Hz) and a high-frequency (100 Hz) phase. The stimulation intensity was set at 1 mA. This taVNS treatment was applied for 30 minutes per day over a period of two weeks.

Following FD model establishment, mice in the taVNS and *Gata3*^∆mac^ + taVNS groups received taVNS treatment. Under 1% isoflurane inhalation anesthesia, two electrodes with opposite magnetic polarities were placed on the inner and outer surfaces of bilateral auricular conchae to ensure current penetration into deep tissues, particularly the auricular vagus nerve (Fig 2A). Physiological saline was applied between electrodes and skin to enhance conductivity.

taVNS was administered daily using a HANS-100A electroacupuncture apparatus for 30 minutes per session over 2 consecutive weeks. The stimulation parameters were: sparse-dense wave pattern, frequency 2/100 Hz, and intensity 1 mA [30] (Fig 2B).

### 2.4 3-Hour food intake behavioral assessment

To evaluate gastric motility and feeding behavior, 3-hour food intake was measured in all experimental groups. Mice were fasted for 12 hours before the experiment, and on the following day, each mouse was provided with 30g of standard pellet feed for 3 hours. After 3 hours, the remaining feed weight was measured. The calculation was performed according to the following formula: 3-hour food intake (g) = 30 (g) – remaining feed (g). Measurements were conducted once before modeling, once after modeling, and once after intervention, and 3-hour food intake was compared among all groups. All measurements were performed at the same time of day to minimize circadian rhythm influences on feeding behavior. To avoid confounding from fasting-induced pathway activation, the identical 12h fasting and 3h refeeding protocol was uniformly applied to all groups at each time point, ensuring balanced baseline conditions across comparisons.

### 2.5 Western blot

Duodenal mucosa samples were homogenized in RIPA lysis buffer containing protease inhibitors and centrifuged at 12,000 rpm for 15 minutes at 4°C. Total protein concentration was determined using the BCA protein assay kit. Equal amounts of protein (40 μg) were separated by 10% SDS-PAGE and transferred to PVDF membranes. After blocking with 5% non-fat milk for 1 hour at room temperature, membranes were incubated overnight at 4°C with primary antibodies against ZO-1 (antibody: Proteintech/21773–1-AP; dilution 1:1000) and Actin (antibody: Abcam/ab8226; dilution 1:5000) as loading control. Following washing, membranes were incubated with HRP-conjugated secondary antibodies (1:5000 dilution) for 1 hour at room temperature. Protein bands were visualized using Enhanced chemiluminescence (ECL) detection reagent and quantified by densitometric analysis. ZO-1 protein expression levels were normalized to Actin and expressed as relative fold changes compared to the control group.

### 2.6 Real-time quantitative PCR analysis

Real-time quantitative PCR was employed to detect mRNA expression levels of target genes. Total RNA was extracted from duodenal mucosal tissues using the SPARKeasy tissue/cell RNA rapid extraction kit. Duodenal mucosal tissues were cut into small fragments and homogenized with RLT Plus lysis buffer, followed by genomic DNA removal using DNA elimination columns, ethanol precipitation with 70% ethanol, purification through RA adsorption columns, and final elution with RNase-free H2O to obtain purified RNA. RNA concentration and purity were assessed using ultraviolet spectrophotometry (A260/A280 ratio between 1.8–2.0).

Reverse transcription was performed using a two-step method, starting with gDNA digestion at 42°C for 2 minutes, followed by cDNA first-strand synthesis using 1 μg total RNA at 25°C for 5 minutes, 55°C for 15 minutes, and 85°C for 2 minutes. The real-time quantitative PCR reaction system consisted of 20 μL total volume containing 10 μL SYBR Green Master Mix (2×), 1 μL cDNA template, 0.5 μL each of forward and reverse primers (10 μM), and 7.5 μL ddH2O. PCR cycling conditions were: initial denaturation at 95°C for 5 minutes, followed by 40 cycles of denaturation at 95°C for 10 seconds and annealing/extension at 60°C for 30 seconds, with final melting curve analysis (60–95°C) to verify product specificity.

Target genes and primer sequences were as follows: *Nos2*: F: 5’-CCGAAGCAAACATCACATTCA-3,’ R: 5’-GGTCTAAAGGCTCCGGGCT-3’ *Arg1*: F: 5’-GTTCCCAGATGTACCAGGATTC-3,’ R: 5’-CGATGTCTTTGGCAGATATGC-3’ UCP2: F: 5’-GGCACAGAAGTGTTCCATAAAGT-3,’ R: 5’-GAGGCAGGGCTTCCGATAG-3’ GATA3: F: 5’-CCAGGCAAGATGAGAAAGAGTG-3,’ R: 5’-ATAGGGCGGATAGGTGGTAATG-3’ GAPDH (reference): F: 5’-TCAAGAAGGTGGTGAAGCAG-3,’ R: 5’-AGGTGGAAGAATGGGAGTTG-3’

Relative expression levels of target genes were calculated using the 2^-ΔΔCt^ method with GAPDH as the reference gene for normalization. Each sample was analyzed in triplicate, and results were expressed as fold changes relative to the control group.

### 2.7 ELISA

ELISA kits were used to detect the concentrations of TNF-α and IL-10 in serum as well as IL-1β and TGF-β1 in duodenal mucosal homogenates..Blood samples were collected after a 12-hour fast following the conclusion of the intervention. 100 μL of standards and samples (serum or diluted tissue supernatants) were added to antibody-coated 96-well plates and incubated at 37°C for 2 hours. After washing, biotinylated detection antibodies were added and incubated at 37°C for 1 hour. Following another wash, streptavidin-HRP conjugate was added and incubated at 37°C for 30 minutes. TMB substrate solution was then added and incubated at room temperature for 15 minutes before stopping the reaction. Absorbance was measured at 450 nm using a microplate reader. For duodenal tissue, approximately 100 mg of mucosal scrapings were accurately weighed and homogenized in 900 µL of normal saline (1:9, w/v) using a tissue grinder. The homogenates were centrifuged at 3000 rpm for 10 min at 4°C, and the supernatants were collected. Prior to assay, tissue supernatants were diluted 2‑fold with the universal diluent provided in the kit.

Standard curves were constructed using serial dilutions of recombinant cytokine standards. Sample concentrations were calculated by interpolation from standard curves using four-parameter logistic curve fitting. For tissue cytokines, the calculated concentrations were multiplied by the dilution factor of 2, and total protein concentrations in tissue supernatants were determined by BCA assay for normalization; final results were expressed as pg/mg protein for duodenal cytokines, while serum cytokine levels were expressed as pg/mg. All samples were analyzed in duplicate.

### 2.8 Statistical analysis

All data were analyzed using GraphPad Prism 10.0. For behavioral parameters (*n* = 6 per group), normality was confirmed via Shapiro‑Wilk tests, and group differences were assessed by one‑way ANOVA with Tukey’s post‑hoc test; F‑values, degrees of freedom, and exact p‑values are provided in the Results section or figure legends. For molecular assays (*n* = 3 per group), data are presented as exploratory, with individual values displayed; formal normality tests were not performed due to the small sample size. Effect sizes (partial *η*²) are reported only for behavioral data where sample sizes permit reliable estimation. All p‑values from multiple comparisons were adjusted using Tukey’s method. Differences were considered significant at *p < 0.05, **p < 0.01, and ***p < 0.001.

## 3. Results

### 3.1 TaVNS restores feeding behavior in a GATA3-dependent manner

**Fig 3:**
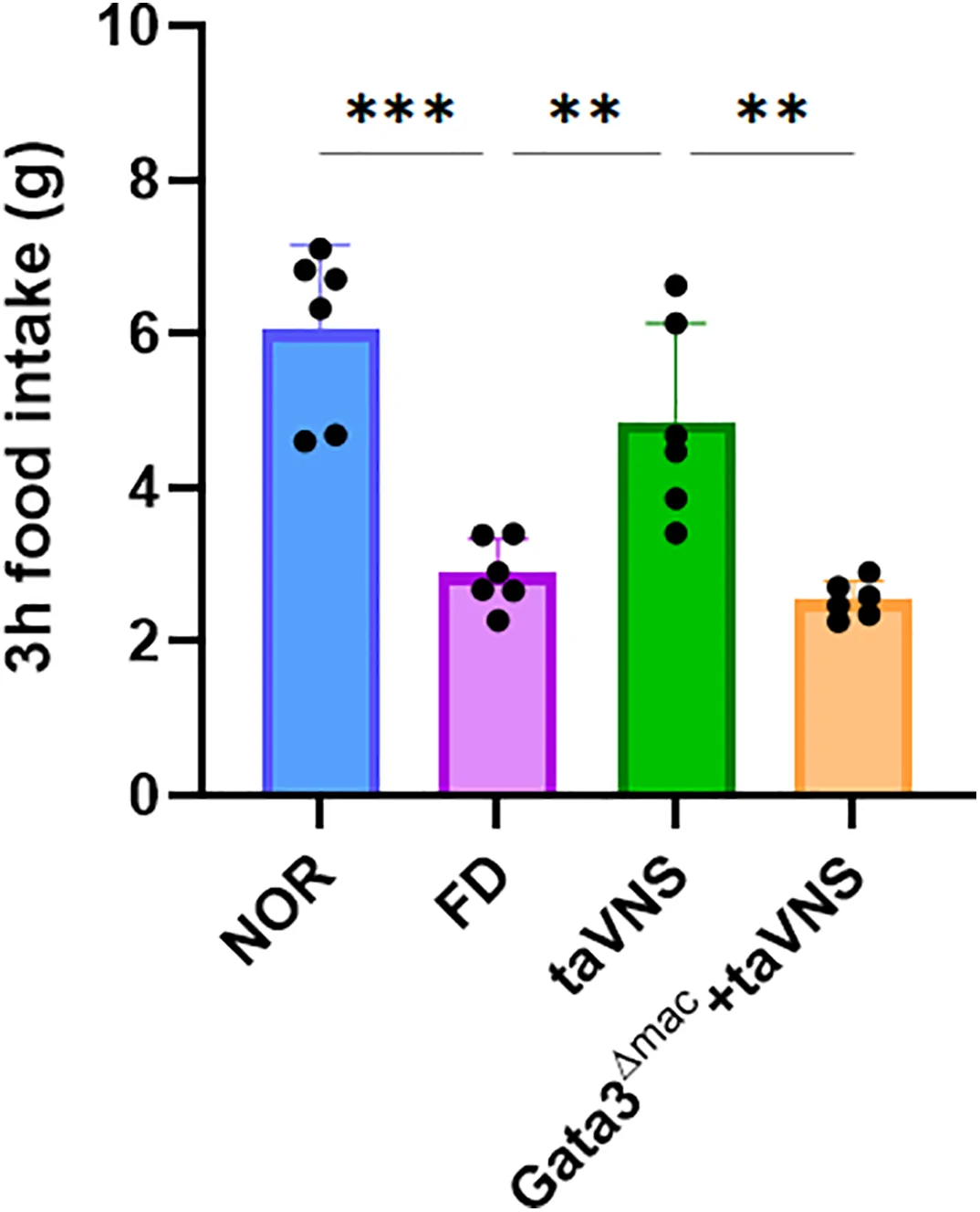
Effect of taVNS treatment on 3-hour food intake in FD mice. One-way ANOVA: *F*(3, 20) = 21.28, *p <* 0.001, partial *η*² = 0.76; Tukey’s post‑hoc adjusted p-values are indicated by asterisks. Data are presented as mean ± SD (*n* = 6 per group). ***p < 0.001, **p < 0.01.

To evaluate the therapeutic effect of taVNS on functional dyspepsia, we examined the feeding behavior of mice. The results showed that compared with the NOR group, the 3-hour food intake of FD group mice was significantly reduced (*p* < 0.001), confirming reduced post-fasting food intake in the model group. After taVNS treatment, the food intake of mice was significantly recovered compared to the FD group (*p* < 0.01), but remained lower than normal levels. However, when taVNS treatment was performed in *Gata3*^∆mac^ + taVNS group, the improvement effect on food intake was attenuated (*p* < 0.01), suggesting that GATA3 plays an important role in taVNS-mediated improvement of feeding function (Fig 3).

### 3.2 TaVNS attenuates duodenal inflammation in FD mice in a GATA3-dependent manner

**Fig 4:**
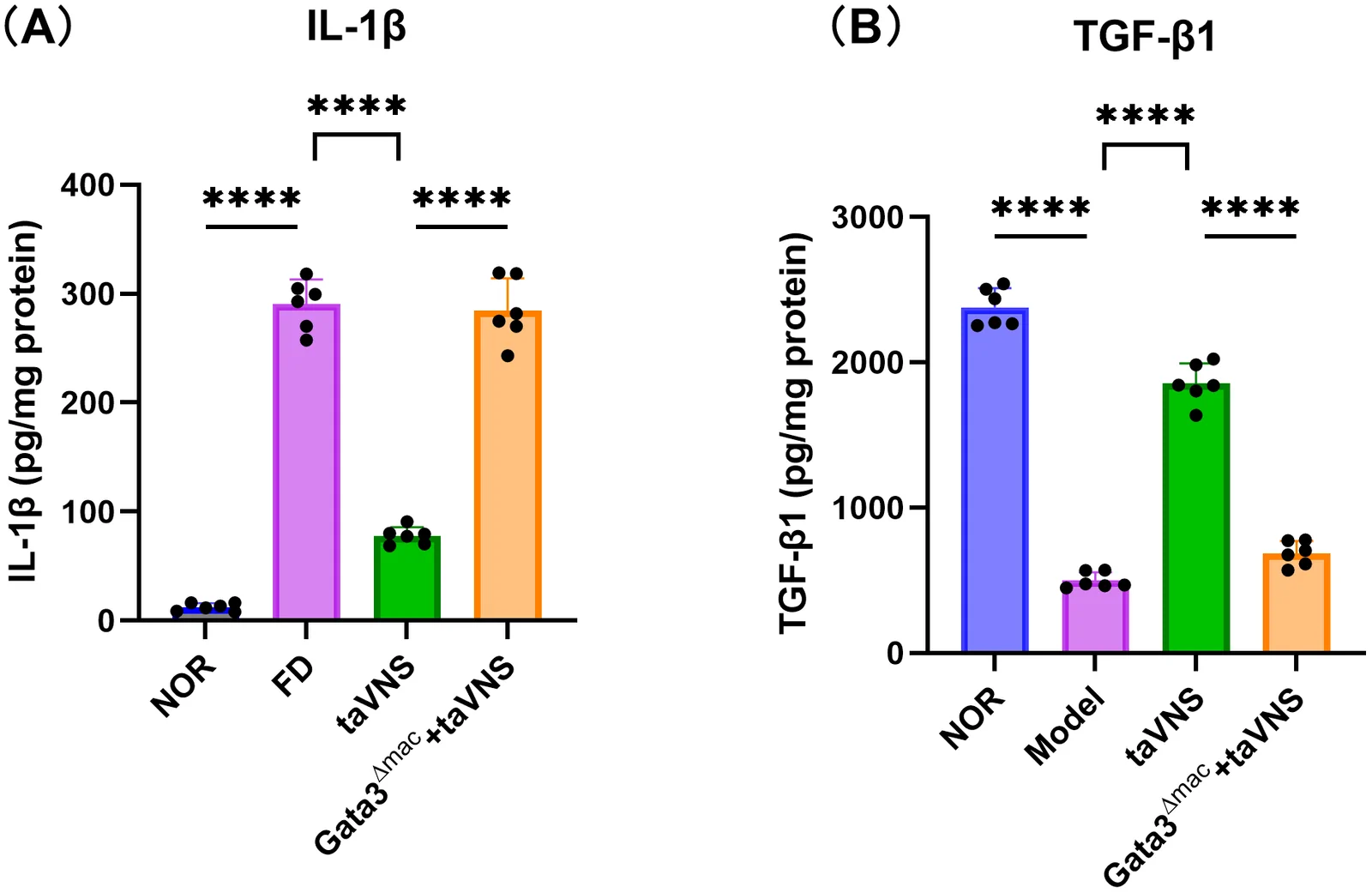
TaVNS modulates duodenal cytokine levels in FD mice in a GATA3-dependent manner. (A) IL-1β: One-way ANOVA: F(3, 20) = 334.1, partial *η*² = 0.98, *p* < 0.0001. (B) TGF-β1: One-way ANOVA: *F*(3, 20) = 428.6, partial *η*² = 0.98, *p* < 0.0001. Tukey’s post‑hoc adjusted p-values are indicated by asterisks. Data are presented as mean ± SD (*n* = 6 per group). ****p < 0.0001.

To investigate the peripheral inflammatory microenvironment, we measured the levels of pro-inflammatory cytokine IL-1βand anti-inflammatory cytokine TGF-β1 in duodenal mucosal homogenates via ELISA. IL-1β levels were markedly elevated in the FD group compared with the NOR group *(p* < 0.001), whereas taVNS treatment significantly reduced IL-1β concentrations (*p* < 0.001; Fig 4A). Conversely, duodenal TGF-β1 levels were dramatically decreased in FD mice versus NOR mice (*p* < 0.001), and this reduction was largely reversed by taVNS treatment (*p* < 0.001; Fig 4B). Notably, the protective effects of taVNS on both IL-1βand TGF-β1 levels were abrogated in the *Gata3*^∆mac^ + taVNS group, as the cytokine profiles in these mice were comparable to those of the untreated FD group (both *p* < 0.001). These results indicate that taVNS effectively alleviates FD-associated duodenal inflammation, and this immunomodulatory effect is critically dependent on GATA3.

### 3.3 TaVNS partially elevates suppressed UCP2 and GATA3 expression in FD mice

**Fig 5:**
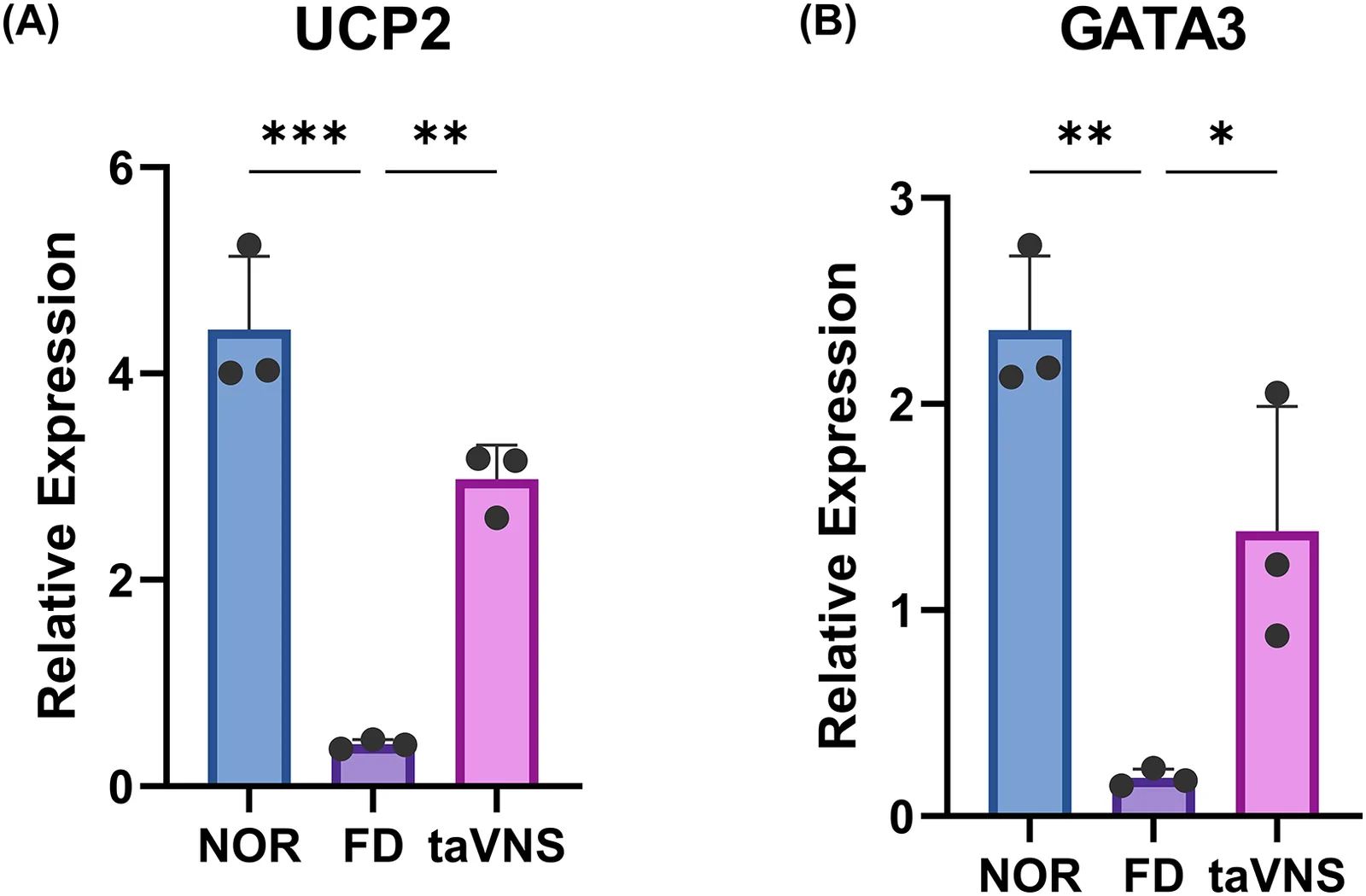
TaVNS partially restores UCP2 and GATA3 expression in FD mice (exploratory, *n* = 3 per group). (A) Relative expression of *Ucp2* mRNA detected by qPCR. One‑way ANOVA: *F*(2, 6) = 60.92, *p* < 0.001, partial *η*² = 0.95. (B) Relative expression of Gata3 mRNA detected by qPCR. One‑way ANOVA: *F*(2, 6) = 13.65, *p* < 0.01, partial *η*² = 0.82. Tukey’s post‑hoc adjusted p‑values are indicated by asterisks. Data are presented as mean ± SD. *p < 0.05, **p < 0.01, ***p < 0.001.

To investigate the molecular mechanisms of taVNS, we examined the expression levels of key regulatory factors GATA3 and UCP2. qPCR results showed that compared with the NOR group, the relative expression of *Gata3* mRNA was significantly downregulated in the FD group (*p* < 0.01), while taVNS treatment significantly increased *Gata3* mRNA expression relative to FD group without recovering to normal levels (*p* < 0.05); Fig 5A). Similarly, UCP2 expression was significantly decreased in the FD group compared to the NOR group (*p* < 0.001), and taVNS treatment increased UCP2 expression compared with FD mice (*p* < 0.01; Fig 5B), partially restoring its expression level. These results demonstrate that taVNS partially restores the expression of UCP2 and GATA3 suppressed by FD lesions.

### 3.4 TaVNS promotes M2 macrophage polarization in a GATA3-dependent manner

**Fig 6:**
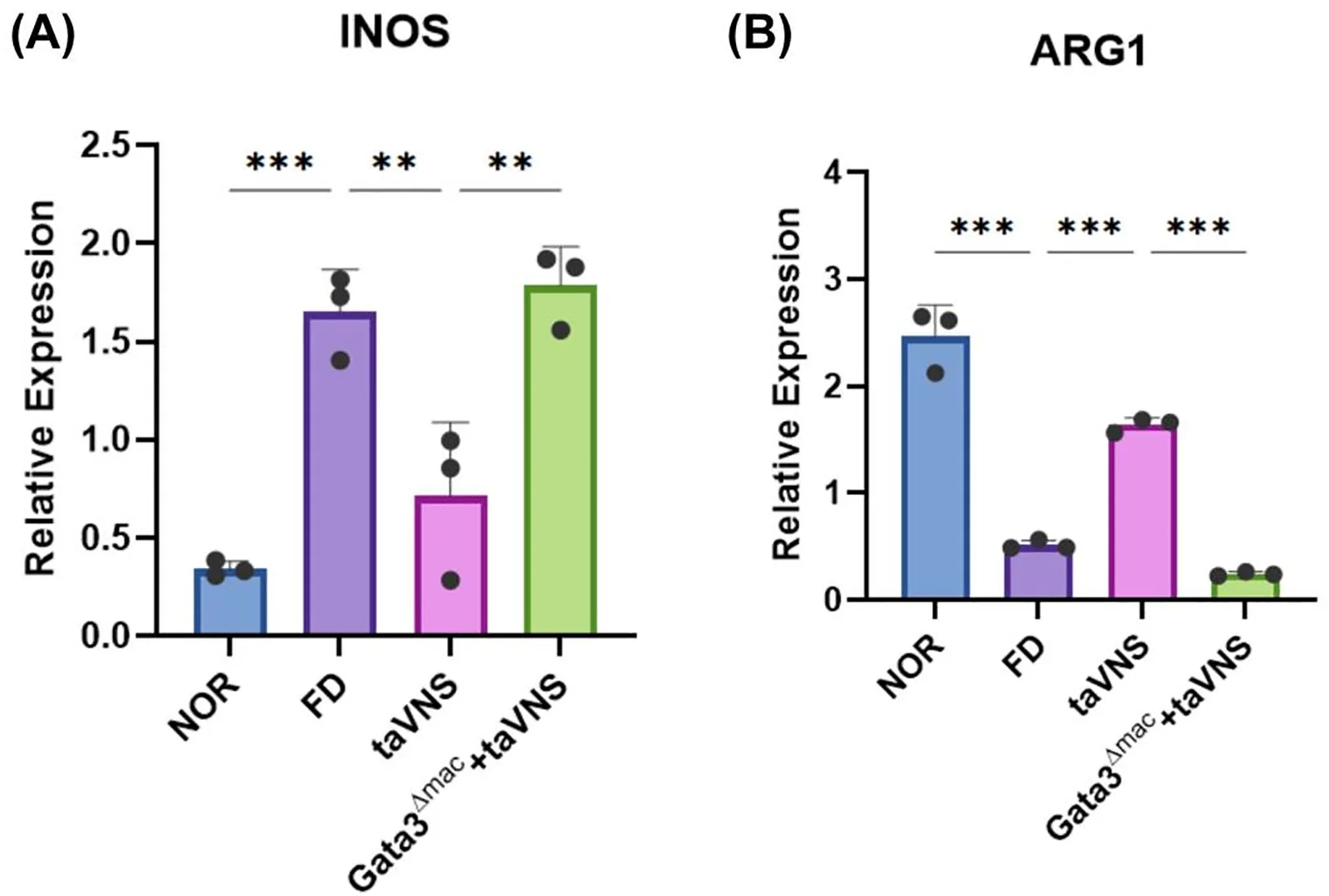
TaVNS promotes M2 macrophage polarization (exploratory, *n* = 3 per group). (A) Relative expression of *INOS* **mRNA detected by qPCR.** One-way ANOVA: *F*(3, 8) = 26.02, *p* < 0.0001, partial *η*² = 0.91.(B) Relative expression of *Arg1* mRNA detected by qPCR. One‑way ANOVA: *F*(3, 8) = 139.7, *p* < 0.001, partial *η*² = 0.98.Tukey’s post‑hoc adjusted p‑values are indicated by asterisks. Data are presented as mean ± SD.**p < 0.01, ***p < 0.001.

To elucidate the regulatory effects of taVNS on macrophage polarization, we examined the specific markers of M1/M2 macrophages. The results showed that compared to the NOR group, the relative mRNA expression of iNOS, an M1 macrophage marker, was upregulated in the FD group (*p* < 0.001; Fig 6A), while the M2 marker *Arg1* expression was significantly downregulated (*p* < 0.001; Fig 6B), indicating that macrophages polarized toward the pro-inflammatory M1 phenotype in FD state, with impaired anti-inflammatory M2 macrophage function.

TaVNS treatment significantly reversed this pathological polarization process: iNOS expression was significantly downregulated compared to the FD group (*p* < 0.01), while *Arg1* expression was upregulated compared to the FD group (*p* < 0.001), suggesting that taVNS can effectively suppress M1 macrophage activation and promote M2 macrophage polarization.

However, in *Gata3*^∆mac^ + taVNS mice treated with taVNS, this beneficial polarization regulatory effect was attenuated: iNOS expression remained significantly higher than the taVNS group (*p* < 0.01), and *Arg1* expression remained significantly lower than the taVNS group (*p* < 0.001), confirming that GATA3 is a key transcription factor for taVNS regulation of macrophage M1/M2 polarization balance.

### 3.5 TaVNS ameliorates intestinal barrier dysfunction and inflammatory responses in FD mice via GATA3-dependent mechanisms

**Fig 7:**
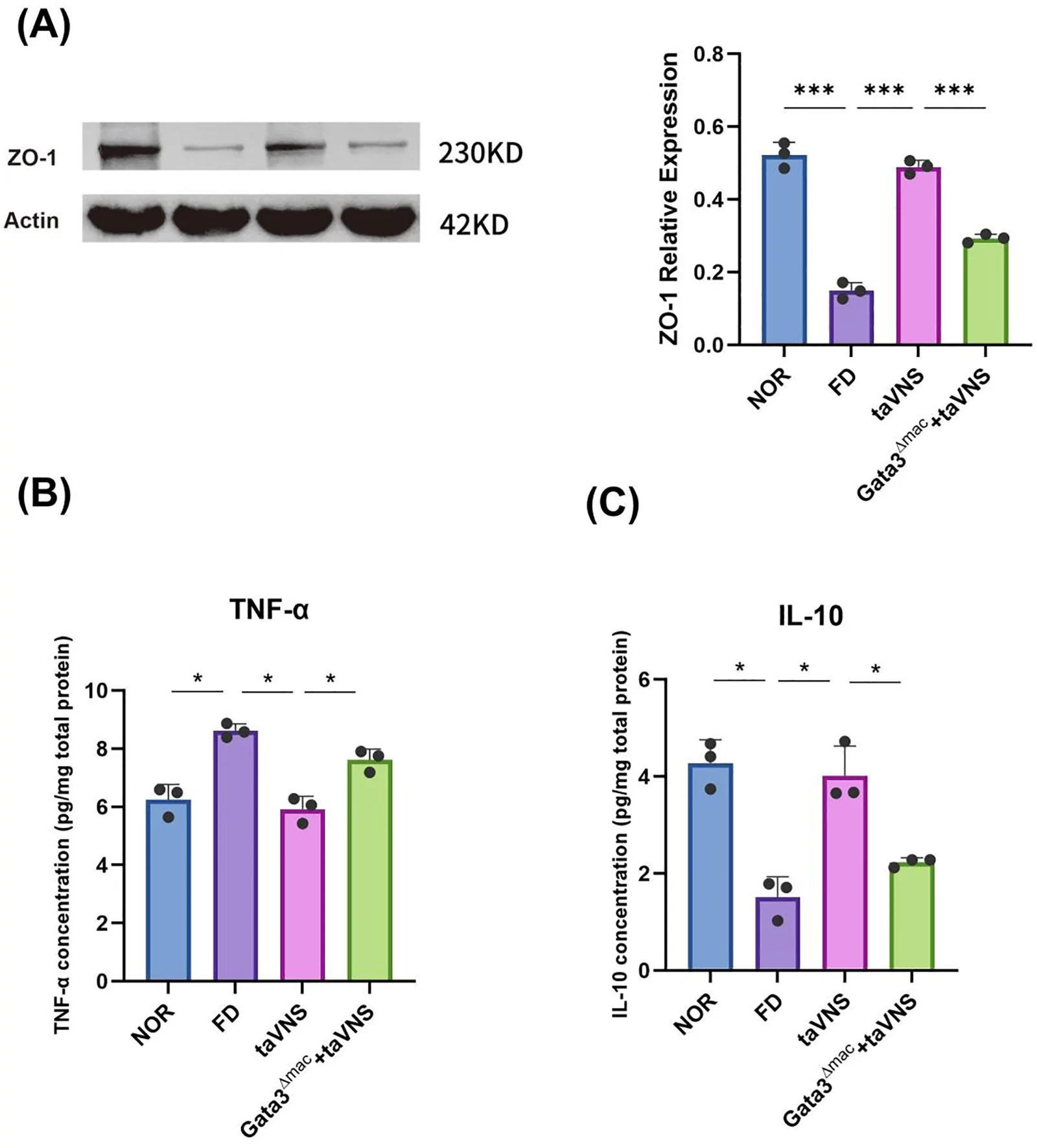
TaVNS modulates ZO-1 and inflammatory cytokine expression in FD mice (exploratory, *n* = 3 per group). (A) Representative Western blot images and quantitative analysis of ZO-1 protein expression. One-way ANOVA: *F*(3, 8) = 169.2, *p* < 0.001, partial *η*² = 0.98.(B) Serum TNF-α concentrations measured by ELISA. One-way ANOVA: *F*(3, 8) = 28.17, *p* < 0.001, partial *η*² = 0.91.(C) Serum IL‑10 levels determined by ELISA. One-way ANOVA: *F*(3, 8) = 27.72, *p* < 0.001, partial *η*² = 0.91. Tukey’s post‑hoc adjusted p-values are indicated by asterisks. Data are presented as mean ± SD. **p < 0.01, ***p < 0.001.

To further validate the protective effects of taVNS on intestinal barrier function and elucidate the underlying mechanisms, we examined the expression of tight junction protein ZO-1 and inflammatory cytokine levels. Western blot analysis revealed that compared to the NOR group, ZO-1 protein expression was significantly downregulated in the FD group (*p* < 0.001; Fig 7A), indicating impaired intestinal tight junctions and compromised barrier function under FD conditions.

taVNS treatment significantly ameliorated these pathological changes: compared to the FD group, ZO-1 protein expression was significantly upregulated (*p* < 0.001), suggesting that taVNS effectively upregulated the expression of tight junction protein ZO-1, indicating alleviated tight junction injury associated with intestinal barrier damage.

Regarding inflammatory cytokine levels, compared to the FD group, taVNS treatment significantly reduced the concentration of pro-inflammatory cytokine TNF-α (*p* < 0.01; Fig 7B) while significantly increasing the level of anti-inflammatory cytokine IL-10 (*p* < 0.01; Fig 7C), indicating that taVNS exerts anti-inflammatory protective effects by modulating the pro-inflammatory/anti-inflammatory cytokine balance.

However, in Gata3^∆mac^ + taVNS mice receiving taVNS treatment, these beneficial effects were attenuated: ZO-1 protein expression remained significantly lower than the taVNS group (*p* < 0.001), TNF-α concentration was re-elevated (*p* < 0.01), and IL-10 levels were decreased (*p* < 0.01), further confirming the crucial role of GATA3 in taVNS-mediated regulation of intestinal barrier function and inflammatory responses.

## 4. Discussion

Our findings demonstrate that taVNS significantly ameliorates feeding behavior in FD mice, with this therapeutic effect closely associated with marked alterations in macrophage polarization states. Specifically, taVNS treatment promotes the conversion of pro-inflammatory M1 macrophages to anti-inflammatory and reparative M2 macrophages, a polarization process accompanied by coordinated increases in UCP2 and GATA3 expression relative to untreated FD mice.

At the duodenal mucosal level, taVNS significantly decreased the elevated IL-1β levels in FD mice while concurrently increasing the suppressed TGF-β1 levels, and these effects were largely abrogated in *Gata3*^∆mac^ + taVNS mice, indicating that GATA3 is essential for taVNS-mediated mucosal immune regulation. More importantly, our findings that taVNS concurrently increased the expression of UCP2 and GATA3 relative to FD model mice and achieved partial restoration suggest a potential functional coupling between them. We speculate that the metabolic reprogramming mediated by UCP2 may act as an upstream event facilitating the transcriptional activity of GATA3. Specifically, UCP2, by regulating mitochondrial function, could influence the intracellular levels of metabolic intermediates that serve as cofactors for epigenetic enzymes, thereby altering chromatin accessibility and creating a favorable environment for GATA3 to drive M2 gene expression. This proposed ‘metabolic-transcriptional’ regulatory axis provides a potential mechanistic link for our observations. However, it is crucial to acknowledge that this hierarchical relationship remains speculative, as our current data only demonstrate concurrent upregulation without direct evidence of interaction or regulatory sequence. Future studies using macrophage-targeting nanoprobes (e.g., NO-responsive afterglow or CD206-targeted agents) could longitudinally track M1/M2 polarization in vivo, correlating with UCP2/GATA3 dynamics to clarify causality and offer noninvasive biomarkers for taVNS efficacy [31]. This phenotypic transition, characterized by increased *Arg1* and decreased iNOS expression, further promotes gastrointestinal tissue repair-related phenotypes, evidenced by elevated ZO-1 tight junction protein expression that alleviates duodenal epithelial tight junction damage. Simultaneously, taVNS treatment significantly modulates the local inflammatory microenvironment by downregulating pro-inflammatory factor TNF-α expression and upregulating anti-inflammatory factor IL-10 production, thereby establishing an immune homeostasis conducive to tissue repair.

Through GATA3 gene knockout experiments, we further confirmed the central role of GATA3 in taVNS therapeutic effects. GATA3 deficiency attenuated all beneficial effects of taVNS on macrophage polarization, tissue repair, and inflammation regulation, indicating that GATA3 acts as a critical transcription factor mediating taVNS downstream effects for taVNS therapeutic action.

Based on our observation that taVNS upregulated UCP2 and GATA3 expression, we explored the potential regulatory mechanisms of macrophage polarization. UCP2, as a mitochondrial uncoupling protein, plays a crucial role in macrophage metabolic reprogramming. Our study showed that UCP2 expression was significantly downregulated in the FD state, while taVNS treatment increased its expression and achieved partial restoration. Mechanistically, UCP2 affects ATP production efficiency and ROS levels by regulating the degree of mitochondrial respiratory chain uncoupling. Previous studies have shown that mitochondrial ROS production is a key determinant of macrophage polarization, with excessive ROS promoting M1 activation while controlled ROS levels favor M2 polarization [32]. Conversely, moderate expression of UCP2 can maintain mitochondrial functional homeostasis, providing the metabolic basis for oxidative phosphorylation and fatty acid oxidation required by M2 macrophages [33]. GATA3, as a key transcription factor, plays important roles in immune cell differentiation and function [16,17]. While GATA3 is well-established as a master regulator of Th2 cell differentiation [34,35], emerging evidence suggests its involvement in macrophage polarization. Our study reveals that GATA3 expression is upregulated during taVNS-induced M2 polarization. The relationship between UCP2 and GATA3 appears to involve metabolic-transcriptional crosstalk, though the precise molecular mechanisms require further investigation. Mitochondrial metabolites such as α-ketoglutarate can influence histone-modifying enzymes, thereby affecting chromatin accessibility and transcription factor activity [36]. This bidirectional regulatory mechanism of metabolism-transcription forms a stable regulatory axis, ensuring the maintenance of M2 macrophage phenotype.

As shown in Fig 4B, *Gata3* mRNA expression was significantly downregulated in the FD group compared with the NOR group (*p* < 0.001), and taVNS treatment significantly increased its expression relative to the FD group (*p* < 0.05). Traditional views hold that vagus nerve stimulation primarily modulates systemic immune responses by activating afferent fibers, which in turn activate descending pathways such as the cholinergic anti‑inflammatory pathway (CAP) through central structures like the solitary tract nucleus in the brainstem [37]. However, taVNS promotes duodenal macrophage polarization toward the M2 phenotype in a manner dependent on the transcription factor GATA3. Given that the auricular branch of the vagus nerve primarily consists of afferent fibers, taVNS likely first activates the central nervous system, subsequently regulating the local intestinal immune microenvironment indirectly through descending pathways including CAP. As a key molecule in this process, GATA3 may receive signals from the taVNS-regulated immune environment, thereby finely tuning the polarization fate of macrophages. This finding deepens our understanding of the complexity of neuro-immune interaction networks and suggests that GATA3 serves as a crucial node linking neuro-regulatory signals to macrophage function. This notion of pathway specificity is strongly supported by our team’s study employing a well-controlled sham protocol in an FD rat model, which demonstrated that active taVNS, but not sham stimulation at a non-vagal auricular site, significantly improved gastric hypersensitivity and motility [38]. This evidence underscores that the benefits of taVNS in FD models are attributable to the engagement of specific neural circuits rather than non-specific cutaneous stimulation or handling effects. taVNS, as a non-invasive neuromodulation technique, has obvious advantages compared to traditional implantable vagus nerve stimulators. taVNS can activate vagal nerve pathways while avoiding surgical risks and achieving better patient compliance [39]. Our study provides a solid mechanistic foundation for the clinical application of taVNS, helping to promote the widespread application of this technology in FD and other functional gastrointestinal diseases. We found that taVNS initiated multilevel mechanisms of intestinal barrier repair by promoting M2 macrophage polarization. M2 macrophages possess powerful tissue repair functions and can secrete various repair factors to promote epithelial healing. Intestinal M2 macrophages are the main source of IL-10, which not only inhibits pro-inflammatory responses but also directly promotes proliferation and differentiation of intestinal epithelial cells [40]. Arginase 1 (*Arg1*) is a characteristic marker of M2 macrophages that produces polyamine substances through arginine metabolism, providing necessary metabolic precursors for cell proliferation. *Arg1* activity is crucial for intestinal epithelial repair and barrier function maintenance [41]. Additionally, M2 macrophages also secrete growth factors such as vascular endothelial growth factor (VEGF) and epidermal growth factor (EGF), promoting angiogenesis and epithelial regeneration [42]. The restoration of tight junction integrity is a key link in intestinal barrier function repair. We observed that taVNS treatment upregulated ZO-1 protein expression. ZO-1, as a key scaffolding protein of tight junctions, directly reflects tight junction integrity through its expression level [43]. ZO-1 is not only a structural protein but also participates in regulating dynamic remodeling and signal transduction of tight junctions [44]. Remodeling of the inflammatory microenvironment is an important prerequisite for barrier repair. We observed that taVNS treatment significantly reduced TNF-α levels and increased IL-10 levels. TNF-α can disrupt the expression and localization of tight junction proteins by activating the NF-κB signaling pathway [45]. Conversely, IL-10 can upregulate the expression of tight junction proteins such as ZO-1 through the STAT3 signaling pathway while inhibiting the production of pro-inflammatory cytokines [46]. This change in pro-inflammatory/anti-inflammatory cytokine balance creates a favorable microenvironment for tissue repair.

The results of *Gata3*^∆mac^ + taVNS group experiments fully demonstrated the contributory role of GATA3 in the taVNS therapeutic mechanism. In GATA3 knockout mice, all beneficial effects of taVNS on feeding behavior, macrophage polarization, intestinal barrier function, and inflammatory responses were attenuated, indicating that GATA3 is a key regulatory node for taVNS to exert therapeutic effects. From the perspective of transcriptional regulatory networks, GATA3 coordinates the expression of multiple genes by directly binding to promoter regions of M2-related genes. GATA3 is a key transcription factor for M2 macrophage differentiation and functional maintenance [41,42]. GATA3 functions as an important transcription factor that can coordinate the expression of multiple genes involved in immune regulation and tissue repair [16,47,48]. The interaction between GATA3 and other transcription factors such as IRF4 may contribute to the maintenance of anti-inflammatory macrophage phenotypes [49,50]. GATA3 synergistically regulates M2 macrophage gene expression programs with KLF4 [49]. IRF4 and GATA3 jointly maintain the anti-inflammatory phenotype of M2 macrophages [50]. Furthermore, GATA3 also participates in epigenetic regulation. GATA3 can recruit histone-modifying enzymes to alter chromatin structure and maintain the open state of M2 gene loci [51]. These characteristics make GATA3 a highly promising therapeutic target.

Limitations of this study should be acknowledged. Our investigation was exclusively conducted in male mice. While this approach reduces hormonal variability, it also limits the generalizability of our findings. Given that sex hormones are known to influence immune responses and that functional dyspepsia exhibits sex differences in prevalence, the therapeutic efficacy and underlying mechanisms of taVNS observed in this study may not be fully extrapolated to female subjects. Additionally, the absence of a sham control limits our ability to attribute the outcomes solely to vagal activation. The mechanistic interplay of the proposed *Ucp2*-GATA3 axis remains incompletely defined. Our data demonstrate that taVNS concurrently increased the expression of UCP2 and GATA3 relative to FD model mice and achieved partial restoration, and that GATA3 deletion was associated with attenuation of several measured outcomes. However, the direct causal or hierarchical relationship between the metabolic regulator UCP2 and the transcription factor GATA3 is still speculative. The current evidence does not rule out the possibility that they operate in parallel pathways rather than a sequential axis. Future studies employing techniques such as co-immunoprecipitation to probe for direct interaction, siRNA-mediated knockdown of UCP2 to observe its impact on GATA3, or GATA3 overexpression studies are necessary to solidify this mechanistic link [45]. Our assessment of macrophage polarization relied primarily on bulk duodenal mRNA expression of iNOS and *Arg1*, which, while widely used as markers, cannot distinguish true phenotype switching in macrophages from changes in other cell types or inflammatory infiltration. Protein-level confirmation and cell-specific analyses (e.g., flow cytometry, macrophage sorting, immunofluorescence co‑localization) were not performed in this study; these are important directions for future research. Similarly, the detailed validation of macrophage‑specific GATA3 knockout (including flow‑sorting and co‑localization) remains to be fully characterized and is therefore reserved for future work. Furthermore, given that our FD model incorporated multiple stressors and the established role of taVNS in modulating central arousal and stress responses, it is plausible that the improvement in feeding behavior was partly mediated by the alleviation of stress-related signals and normalization of central arousal, thereby disrupting the vicious cycle between psychological stress and gastrointestinal dysfunction. Finally, our study primarily focused on local intestinal immune regulation. However, as a neuromodulation technique, taVNS likely engages broader neuro-endocrine-immune networks. The systemic effects and their interplay with the local immune regulation described here warrant further investigation. Notably, our evaluation of intestinal barrier status was limited to detecting tight junction protein ZO-1; intestinal permeability functional tests and duodenal mucosal histological staining were not performed to directly verify barrier integrity, which is a limitation of this study.

## 5. Conclusion

This study identifies partial molecular mechanisms through which bilateral transcutaneous auricular vagus nerve stimulation (taVNS) alleviates functional dyspepsia, outlining empirically observed associations spanning neuromodulation to symptomatic improvement. Measured experimental outcomes demonstrate taVNS elevates *Ucp2*-GATA3 transcript abundance, accompanied by phenotypic shifts toward anti-inflammatory M2 macrophages. At the duodenal mucosa, taVNS reduced IL-1β and restored TGF-β1 levels in a GATA3‑dependent manner. TaVNS partially restores tight-junction ZO‑1 expression to mitigate duodenal epithelial barrier damage and rescues disordered feeding patterns in FD mouse models: intervention upregulated *Arg1* and IL-10 while suppressing iNOS and TNF-α expression, and restored ZO-1 levels, alleviating duodenal tight junction injury.

*Gata3*^∆mac^ + taVNS group experiments confirmed the crucial role of GATA3 in the treatment process. Ablation of GATA3 markedly weakened the protective phenotypes produced by taVNS treatment. While the correlational dataset supports GATA3 serving as a key node within the hypothesized immunometabolic axis, upstream regulatory hierarchies of the *Ucp2*-GATA3 cascade remain unconfirmed. These phenotypic observations establish GATA3 as a plausible therapeutic target for FD intervention, requiring follow-up mechanistic assays to validate the proposed signaling cascade.

## Supporting information

S1 File The blot images are labeled with sample groups and molecular weight markers; unused lanes are marked with “X”. (PDF)

S1 Table Original ELISA data for serum TNF-α and IL-10 (corresponding to Fig 7B and 7C). Values are expressed as pg/mL. Original quantitative data underlying Fig 7. The file contains three sheets: (1) densitometric values for ZO-1 Western blot (corresponding to Figure 7A); (2) serum TNF-α concentrations in pg/mL (corresponding to Figure 7B); and (3) serum IL-10 concentrations in pg/mL (corresponding to Figure 7C).
(XLS)

S2 Table Original data for 3-hour food intake, duodenal IL-1β/TGF-β1, and qPCR Ct values. (corresponding to Figures 3, 4, 5, and 6).
(XLSX)

## References

[1] Ford AC, Mahadeva S, Carbone MF, Lacy BE, Talley NJ. Functional dyspepsia. Lancet. 2020;396(10263):1689–702. doi: 10.1016/S0140-6736(20)30469-4 33049222

[2] Black CJ, Drossman DA, Talley NJ, Ruddy J, Ford AC. Functional gastrointestinal disorders: advances in understanding and management. Lancet. 2020;396(10263):1664–74. doi: 10.1016/S0140-6736(20)32115-2 33049221

[3] Varghese C, Carson DA, Bhat S, Hayes TCL, Gharibans AA, Andrews CN, et al. Clinical associations of functional dyspepsia with gastric dysrhythmia on electrogastrography: a comprehensive systematic review and meta-analysis. Neurogastroenterol Motil. 2021;33(12):e14151. doi: 10.1111/nmo.14151 33830590

[4] Aziz I, Palsson OS, Törnblom H, Sperber AD, Whitehead WE, Simrén M. The prevalence and impact of overlapping Rome IV-diagnosed functional gastrointestinal disorders on somatization, quality of life, and healthcare utilization: a cross-sectional general population study in three countries. Am J Gastroenterol. 2018;113(1):86–96. doi: 10.1038/ajg.2017.421 29134969

[5] Enck P, Aziz Q, Barbara G, Farmer AD, Fukudo S, Mayer EA, et al. Functional dyspepsia. Nat Rev Dis Primers. 2017;3:17081. doi: 10.1038/nrdp.2017.81 29099093

[6] Vanuytsel T, Tack J, Farre R. The role of intestinal permeability in gastrointestinal disorders and current methods of evaluation. Front Nutr. 2021;8:717925. doi: 10.3389/fnut.2021.717925 34513903 PMC8427160

[7] Walker MM, Potter MD, Talley NJ. Tangible pathologies in functional dyspepsia. Best Pract Res Clin Gastroenterol. 2019;101650. doi: 10.1016/j.bpg.2019.101650 31594648

[8] Burns G, Carroll G, Mathe A, Horvat J, Foster P, Walker MM, et al. Evidence for local and systemic immune activation in functional dyspepsia and the irritable bowel syndrome: a systematic review. Am J Gastroenterol. 2019;114(3):429–36. doi: 10.1038/s41395-018-0377-0 30839392

[9] Koloski NA, Jones M, Kalantar J, Weltman M, Zaguirre J, Talley NJ. The brain--gut pathway in functional gastrointestinal disorders is bidirectional: a 12-year prospective population-based study. Gut. 2012;61(9):1284–90. doi: 10.1136/gutjnl-2011-300474 22234979

[10] Hegarty LM, Jones G-R, Bain CC. Macrophages in intestinal homeostasis and inflammatory bowel disease. Nat Rev Gastroenterol Hepatol. 2023;20(8):538–53. doi: 10.1038/s41575-023-00769-0 37069320

[11] Viola A, Munari F, Sánchez-Rodríguez R, Scolaro T, Castegna A. The metabolic signature of macrophage responses. Front Immunol. 2019;10:1462. doi: 10.3389/fimmu.2019.01462 31333642 PMC6618143

[12] Koelwyn GJ, Corr EM, Erbay E, Moore KJ. Regulation of macrophage immunometabolism in atherosclerosis. Nat Immunol. 2018;19(6):526–37. doi: 10.1038/s41590-018-0113-3 29777212 PMC6314674

[13] Wculek SK, Dunphy G, Heras-Murillo I, Mastrangelo A, Sancho D. Metabolism of tissue macrophages in homeostasis and pathology. Cell Mol Immunol. 2022;19(3):384–408. doi: 10.1038/s41423-021-00791-9 34876704 PMC8891297

[14] Broche B, Ben Fradj S, Aguilar E, Sancerni T, Bénard M, Makaci F, et al. Mitochondrial protein UCP2 controls pancreas development. Diabetes. 2018;67(1):78–84. doi: 10.2337/db17-0118 29079704

[15] Diano S, Horvath TL. Mitochondrial uncoupling protein 2 (UCP2) in glucose and lipid metabolism. Trends Mol Med. 2012;18(1):52–8. doi: 10.1016/j.molmed.2011.08.003 21917523

[16] Zhu J. GATA3 regulates the development and functions of innate lymphoid cell subsets at multiple stages. Front Immunol. 2017;8:1571. doi: 10.3389/fimmu.2017.01571 29184556 PMC5694433

[17] Hosokawa H, Rothenberg EV. How transcription factors drive choice of the T cell fate. Nat Rev Immunol. 2021;21(3):162–76. doi: 10.1038/s41577-020-00426-6 32918063 PMC7933071

[18] Komal S, Han S-N, Cui L-G, Zhai M-M, Zhou Y-J, Wang P, et al. Epigenetic regulation of macrophage polarization in cardiovascular diseases. Pharmaceuticals (Basel). 2023;16(2):141. doi: 10.3390/ph16020141 37259293 PMC9963081

[19] Farmer AD, Strzelczyk A, Finisguerra A, Gourine AV, Gharabaghi A, Hasan A, et al. International Consensus Based Review and Recommendations for Minimum Reporting Standards in Research on Transcutaneous Vagus Nerve Stimulation (Version 2020). Front Hum Neurosci. 2021;14:568051. doi: 10.3389/fnhum.2020.568051 33854421 PMC8040977

[20] Zhao M, Wang Y, Li L, Liu S, Wang C, Yuan Y, et al. Mitochondrial ROS promote mitochondrial dysfunction and inflammation in ischemic acute kidney injury by disrupting TFAM-mediated mtDNA maintenance. Theranostics. 2021;11(4):1845–63. doi: 10.7150/thno.50905 33408785 PMC7778599

[21] Kang D, Choi Y, Lee J, Park E, Kim IY. Analysis of taVNS effects on autonomic and central nervous systems in healthy young adults based on HRV, EEG parameters. J Neural Eng. 2024;21(4). doi: 10.1088/1741-2552/ad5d16 38941990

[22] Tan G, Huguenard AL, Donovan KM, Demarest P, Liu X, Li Z, et al. The effect of transcutaneous auricular vagus nerve stimulation on cardiovascular function in subarachnoid hemorrhage patients: a randomized trial. Elife. 2025;13:RP100088. doi: 10.7554/eLife.100088 39786346 PMC11717364

[23] Keute M, Machetanz K, Berelidze L, Guggenberger R, Gharabaghi A. Neuro-cardiac coupling predicts transcutaneous auricular vagus nerve stimulation effects. Brain Stimul. 2021;14(2):209–16. doi: 10.1016/j.brs.2021.01.001 33422683

[24] Ventura-Bort C, Wirkner J, Wendt J, Hamm AO, Weymar M. Establishment of emotional memories is mediated by vagal nerve activation: evidence from noninvasive taVNS. J Neurosci. 2021;41(36):7636–48. doi: 10.1523/JNEUROSCI.2329-20.2021 34281991 PMC8425981

[25] Tan G, Adams J, Donovan K, Demarest P, Willie JT, Brunner P, et al. Does vibrotactile stimulation of the auricular vagus nerve enhance working memory? A behavioral and physiological investigation. Brain Stimul. 2024;17(2):460–8. doi: 10.1016/j.brs.2024.04.002 38593972 PMC11268363

[26] Liang Q, Yan Y, Mao L, Du X, Liang J, Liu J, et al. Evaluation of a modified rat model for functional dyspepsia. Saudi J Gastroenterol. 2018;24(4):228–35. doi: 10.4103/sjg.SJG_505_17 29652029 PMC6080150

[27] Ludwig M, Wienke C, Betts MJ, Zaehle T, Hämmerer D. Current challenges in reliably targeting the noradrenergic locus coeruleus using transcutaneous auricular vagus nerve stimulation (taVNS). Auton Neurosci. 2021;236:102900. doi: 10.1016/j.autneu.2021.102900 34781120

[28] Redgrave J, Day D, Leung H, Laud PJ, Ali A, Lindert R, et al. Safety and tolerability of Transcutaneous Vagus Nerve stimulation in humans; a systematic review. Brain Stimul. 2018;11(6):1225–38. doi: 10.1016/j.brs.2018.08.010 30217648

[29] Yap JYY, Keatch C, Lambert E, Woods W, Stoddart PR, Kameneva T. Critical review of transcutaneous vagus nerve stimulation: challenges for translation to clinical practice. Front Neurosci. 2020;14:284. doi: 10.3389/fnins.2020.00284 32410932 PMC7199464

[30] Owens MM, Jacquemet V, Napadow V, Lewis N, Beaumont E. Brainstem neuronal responses to transcutaneous auricular and cervical vagus nerve stimulation in rats. J Physiol. 2024;602(16):4027–52. doi: 10.1113/JP286680 39031516 PMC11326965

[31] Zhang J, Tang K, Yang Y, Yang D, Fan W. Advanced nanoprobe strategies for imaging macrophage polarization in cancer immunology. Research (Wash D C). 2025;8:0622. doi: 10.34133/research.0622 39990770 PMC11842672

[32] Huang SC-C, Everts B, Ivanova Y, O’Sullivan D, Nascimento M, Smith AM, et al. Cell-intrinsic lysosomal lipolysis is essential for alternative activation of macrophages. Nat Immunol. 2014;15(9):846–55. doi: 10.1038/ni.2956 25086775 PMC4139419

[33] Zheng W, Flavell RA. The transcription factor GATA-3 is necessary and sufficient for Th2 cytokine gene expression in CD4 T cells. Cell. 1997;89(4):587–96. doi: 10.1016/s0092-8674(00)80240-8 9160750

[34] Zhu J, Yamane H, Cote-Sierra J, Guo L, Paul WE. GATA-3 promotes Th2 responses through three different mechanisms: induction of Th2 cytokine production, selective growth of Th2 cells and inhibition of Th1 cell-specific factors. Cell Res. 2006;16(1):3–10. doi: 10.1038/sj.cr.7310002 16467870

[35] Liu P-S, Wang H, Li X, Chao T, Teav T, Christen S, et al. α-ketoglutarate orchestrates macrophage activation through metabolic and epigenetic reprogramming. Nat Immunol. 2017;18(9):985–94. doi: 10.1038/ni.3796 28714978

[36] Bonaz B, Sinniger V, Pellissier S. The vagus nerve in the neuro-immune axis: implications in the pathology of the gastrointestinal tract. Front Immunol. 2017;8:1452. doi: 10.3389/fimmu.2017.01452 29163522 PMC5673632

[37] Han J, Wei W, Wang H, Zhang T, Wang Y, Hou L, et al. Effects of transcutaneous auricular vagus nerve stimulation on gastric hypersensitivity and motility in a rat model of functional dyspepsia. Acupunct Res. 2022;47(6):517–24. doi: 10.13702/j.1000-0607.20220111 35764519

[38] Frangos E, Ellrich J, Komisaruk BR. Non-invasive access to the vagus nerve central projections via electrical stimulation of the external ear: fMRI evidence in humans. Brain Stimul. 2015;8(3):624–36. doi: 10.1016/j.brs.2014.11.018 25573069 PMC4458242

[39] Bain CC, Bravo-Blas A, Scott CL, Perdiguero EG, Geissmann F, Henri S, et al. Constant replenishment from circulating monocytes maintains the macrophage pool in the intestine of adult mice. Nat Immunol. 2014;15(10):929–37. doi: 10.1038/ni.2967 25151491 PMC4169290

[40] Rath M, Müller I, Kropf P, Closs EI, Munder M. Metabolism via arginase or nitric oxide synthase: two competing arginine pathways in macrophages. Front Immunol. 2014;5:532. doi: 10.3389/fimmu.2014.00532 25386178 PMC4209874

[41] Wang Z, Lu Y-L, Zhao W-T, Zhong J, Lin X, Sun Z, et al. Distinct origins and functions of cardiac orthotopic macrophages. Basic Res Cardiol. 2020;115(2):8. doi: 10.1007/s00395-019-0769-3 31897858

[42] Suzuki T. Regulation of the intestinal barrier by nutrients: The role of tight junctions. Anim Sci J. 2020;91(1):e13357. doi: 10.1111/asj.13357 32219956 PMC7187240

[43] Bhat AA, Uppada S, Achkar IW, Hashem S, Yadav SK, Shanmugakonar M, et al. Tight junction proteins and signaling pathways in cancer and inflammation: a functional crosstalk. Front Physiol. 2019;9:1942. doi: 10.3389/fphys.2018.01942 30728783 PMC6351700

[44] Ma TY, Iwamoto GK, Hoa NT, Akotia V, Pedram A, Boivin MA, et al. TNF-alpha-induced increase in intestinal epithelial tight junction permeability requires NF-kappa B activation. Am J Physiol Gastrointest Liver Physiol. 2004;286(3):G367-76. doi: 10.1152/ajpgi.00173.2003 14766535

[45] Zheng L, Kelly CJ, Battista KD, Schaefer R, Lanis JM, Alexeev EE, et al. Microbial-derived butyrate promotes epithelial barrier function through IL-10 receptor-dependent repression of Claudin-2. J Immunol. 2017;199(8):2976–84. doi: 10.4049/jimmunol.1700105 28893958 PMC5636678

[46] Aegerter H, Lambrecht BN, Jakubzick CV. Biology of lung macrophages in health and disease. Immunity. 2022;55(9):1564–80. doi: 10.1016/j.immuni.2022.08.010 36103853 PMC9533769

[47] Wynn TA, Chawla A, Pollard JW. Macrophage biology in development, homeostasis and disease. Nature. 2013;496(7446):445–55. doi: 10.1038/nature12034 23619691 PMC3725458

[48] Satoh T, Takeuchi O, Vandenbon A, Yasuda K, Tanaka Y, Kumagai Y, et al. The Jmjd3-Irf4 axis regulates M2 macrophage polarization and host responses against helminth infection. Nat Immunol. 2010;11(10):936–44. doi: 10.1038/ni.1920 20729857

[49] Lawrence T, Natoli G. Transcriptional regulation of macrophage polarization: enabling diversity with identity. Nat Rev Immunol. 2011;11(11):750–61. doi: 10.1038/nri3088 22025054

[50] Yamashita M, Hirahara K, Shinnakasu R, Hosokawa H, Norikane S, Kimura MY, et al. Crucial role of MLL for the maintenance of memory T helper type 2 cell responses. Immunity. 2006;24(5):611–22. doi: 10.1016/j.immuni.2006.03.017 16713978

[51] Hesampour F, Tshikudi DM, Bernstein CN, Ghia J-E. Exploring the efficacy of Transcutaneous Auricular Vagus nerve stimulation (taVNS) in modulating local and systemic inflammation in experimental models of colitis. Bioelectron Med. 2024;10(1):29. doi: 10.1186/s42234-024-00162-5 39648211 PMC11626753

