# Supplementary material for: Mechanisms of transcutaneous bilateral auricular vagus nerve stimulation in treating functional dyspepsia through promoting M2 macrophage polarization
Source: PLoS One. 2026 Aug 21;21(8):e0328670. doi: 10.1371/journal.pone.0328670 (PMC13496460; doi:10.1371/journal.pone.0328670)
Supplement: Supplementary file 1 [file pone.0328670.s001.pdf]

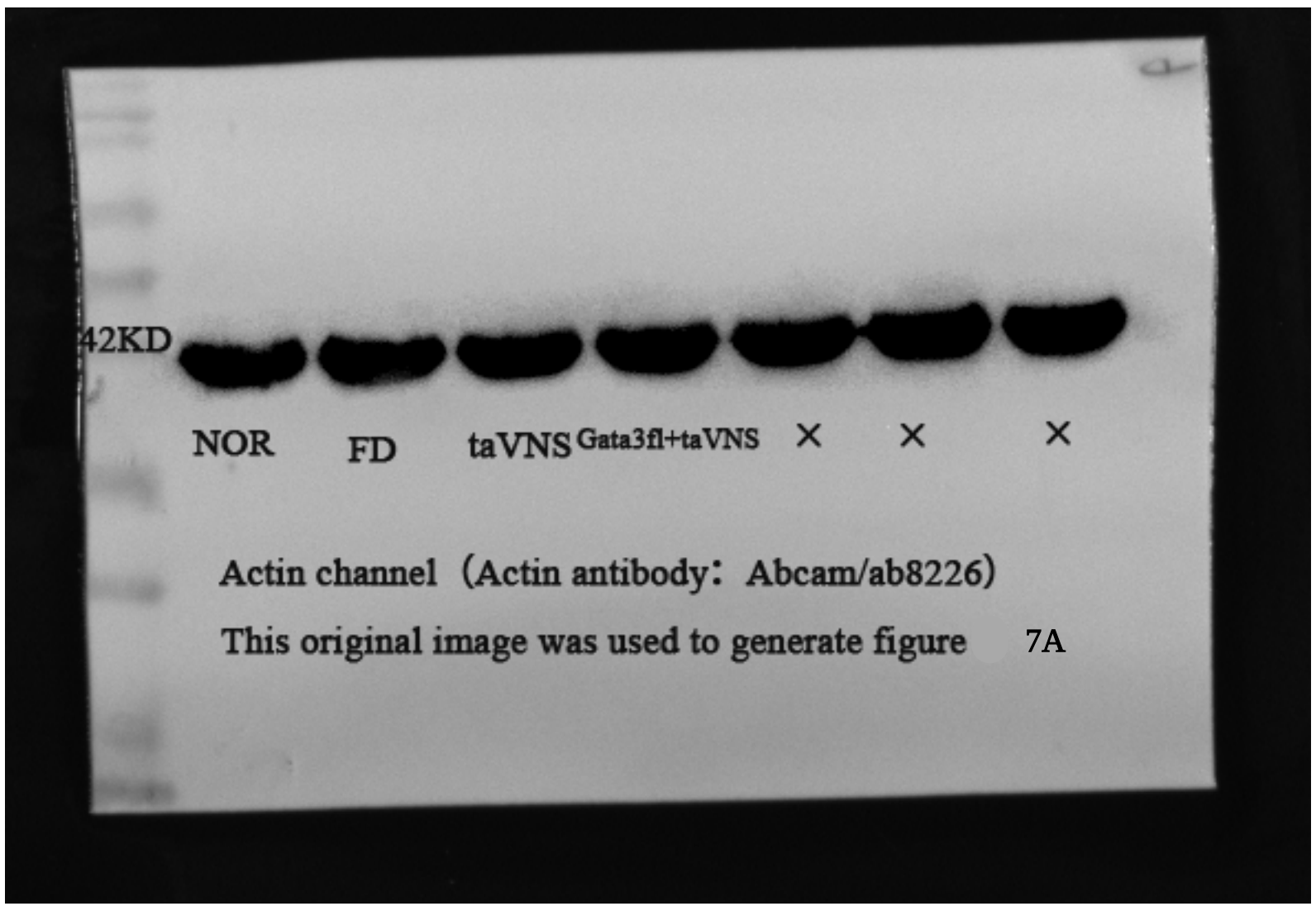
42KD
NOR
FD
taVNS
Gata3fl+taVNS
×
×
×
Actin channel（Actin antibody：Abcam/ab8226）
This original image was used to generate figure 7A

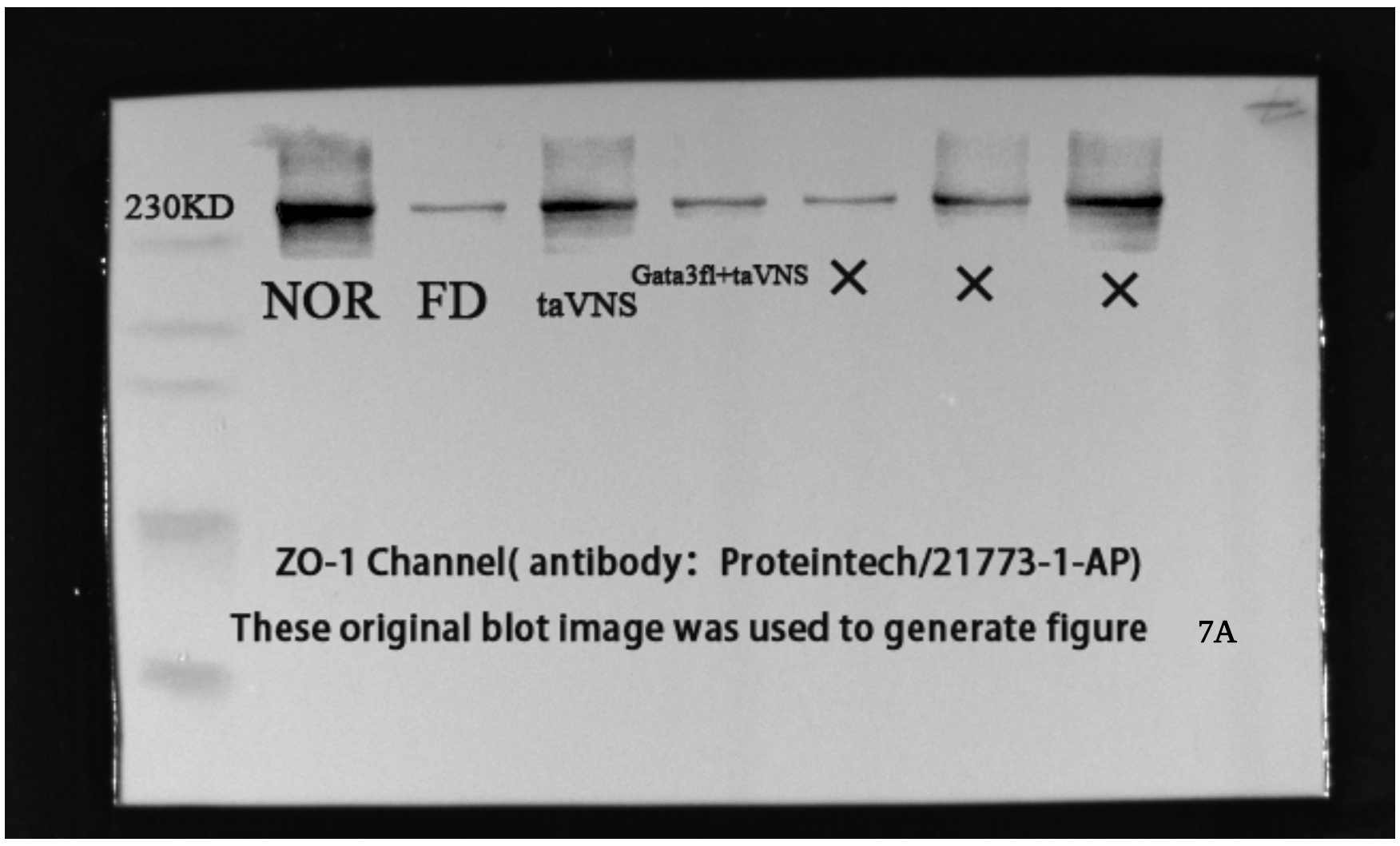
230KD
NOR
FD
taVNS
Gata3fl+taVNS
ZO-1 Channel(antibody：Proteintech/21773-1-AP)
These original blot image was used to generate figure 7A
